# Clinical validation of genetic variants associated with *in vitro* chemotherapy-related lymphoblastoid cell toxicity

**DOI:** 10.18632/oncotarget.17726

**Published:** 2017-05-09

**Authors:** Peter A. Fasching, Lothar Häberle, Brigitte Rack, Liang Li, Alexander Hein, Arif B. Ekici, Andre Reis, Michael P. Lux, Julie M. Cunningham, Matthias Ruebner, Gergory Jenkins, Brooke Fridley, Andreas Schneeweiss, Hans Tesch, Werner Lichtenegger, Tanja Fehm, Georg Heinrich, Mahdi Rezai, Matthias W. Beckmann, Wolfgang Janni, Richard M. Weinshilboum, Liewei Wang

**Affiliations:** ^1^ Department of Gynecology and Obstetrics, Erlangen University Hospital, Friedrich-Alexander-University Erlangen-Nuremberg, Comprehensive Cancer Center Erlangen-EMN, Erlangen, Germany; ^2^ Department of Medicine, Division of Hematology/Oncology, University of California at Los Angeles, David Geffen School of Medicine, Los Angeles, CA, USA; ^3^ Department of Gynecology and Obstetrics, Biostatistics Unit, Erlangen University Hospital, Friedrich-Alexander-University Erlangen-Nuremberg, Erlangen, Germany; ^4^ Department of Gynecology and Obstetrics, Ludwig-Maximilians-University Munich, Munich, Germany; ^5^ Division of Clinical Pharmacology, Department of Molecular Pharmacology and Experimental Therapeutics, Mayo Clinic College of Medicine, Mayo Medical School-Mayo Foundation, Rochester, MN, USA; ^6^ Department of Oncology, Institute of Medicinal Biotechnology, Chinese Academy of Medical Sciences & Peking Union Medical College, Beijing, China; ^7^ Institute of Human Genetics, Erlangen University Hospital, Friedrich-Alexander-University Erlangen-Nuremberg, Erlangen, Germany; ^8^ Department of Laboratory Medicine & Pathology, Mayo Clinic, Rochester, MN, USA; ^9^ Department of Health Sciences Research, Division of Biomedical Statistics and Informatics, Mayo Clinic, Rochester, MN, USA; ^10^ Department of Biostatistics, University of Kansas Medical Center, Kansas City, KS, USA; ^11^ Division of Gynecologic Oncology, National Center for Tumor Diseases, University Hospital Heidelberg, Heidelberg, Germany; ^12^ Department of Oncology, Onkologie Bethanien, Frankfurt, Germany; ^13^ Department of Gynecology and Obstetrics, Charité University Hospital, Campus Virchow, Berlin, Germany; ^14^ Department of Gynecology and Obstetrics, Düsseldorf University Hospital, Heinrich Heine University, Düsseldorf, Germany; ^15^ Department of Gynecologic Oncology, Schwerpunktpraxis für Gynäkologische Onkologie, Fürstenwalde, Germany; ^16^ Department of Breast Diseases, Breast Center of Düsseldorf, Luisenkrankenhaus, Düsseldorf, Germany; ^17^ Department of Gynecology and Obstetrics, Ulm University Hospital, Ulm, Germany

**Keywords:** chemotherapy, neutropenia, leukopenia, SNP, polymorphism

## Abstract

Hematotoxicity is one of the major side effects of chemotherapy. The aim of this study was to examine the association between single nucleotide polymorphisms (SNPs) and hematotoxicity in breast cancer patients in a subset of patients of the SUCCESS prospective phase III chemotherapy study. All patients (n = 1678) received three cycles of 5-fluorouracil, epirubicin, and cyclophosphamide (FEC) followed by three cycles of docetaxel or docetaxel/gemcitabine, depending on randomization. Germline DNA was genotyped for 246 SNPs selected from a previous genome-wide association study (GWAS) in a panel of lymphoblastoid cell lines, with gemcitabine toxicity as the phenotype. All SNPs were tested for their value in predicting grade 3 or 4 neutropenic or leukopenic events (NLEs). Their prognostic value in relation to overall survival and disease-free survival was also tested.

None of the SNPs was found to be predictive for NLEs during treatment with docetaxel/gemcitabine. Two SNPs in and close to the *PIGB* gene significantly improved the prediction of NLEs after FEC, in addition to the factors of age and body surface area. The top SNP (rs12050587) had an odds ratio of 1.38 per minor allele (95% confidence interval, 1.17 to 1.62). No associations were identified for predicting disease-free or overall survival.

Genetic variance in the *PIGB* gene may play a role in determining interindividual differences in relation to hematotoxicity after FEC chemotherapy.

## INTRODUCTION

### General introduction, clinical importance

Myelotoxicity is one of the major side effects of chemotherapy, and it leads to anemia, thrombopenia, and leukopenia. Severe leukopenia or neutropenia can be complicated by life-threatening infections (febrile neutropenia, FN) that require hospitalization, isolation, and broad-spectrum antibiotic therapy [1]. Febrile neutropenia is associated with a high mortality rate, at 5–20% [2–8]. Dose reductions and treatment delays in patients with neutropenic or leukopenic events (NLEs) may also further compromise the prognosis for these patients [9]. The sequelae of FN are serious consequences of the treatment, but administering granulocyte colony-stimulating factor (G-CSF) has been shown to be effective in reducing the rate of FN by 50% [10–15]. In view of the effectiveness and safety of G-CSF, it has been widely incorporated into clinical practice on the basis of individual patients’ risk for developing FN. Patients who receive a chemotherapy regimen with a greater than 20% risk for FN, and those receiving chemotherapy with a risk from 10% to 20% but with additional risk factors as well, are considered to be candidates for prophylactic treatment with G-CSF [14, 16, 17]. Special attention needs to be given to risk factors for FN and NLEs. This is of prime importance in breast cancer patients who are receiving chemotherapy, since one of the most widely used regimens (anthracyclines followed by sequential use of taxanes) is considered to have an intermediate level of risk for FN, at 10–20%.

Age has been consistently identified as a risk factor for FN and NLEs [18–25], and other risk factors such as advanced disease stage, previous episodes of NLEs, and other comorbidities have been also been reported. For breast cancer patients receiving anthracycline-containing chemotherapy — with 5-fluorouracil, epirubicin, and cyclophosphamide (FEC) — models have been developed for predicting neutropenia, FN, or a need for dose-intensity reduction on the basis of a baseline neutrophil and lymphocyte count [23]. Another study including breast cancer patients identified older age, lower weight, higher planned dose intensity, vascular comorbidity, a low baseline white blood cell count, and elevated baseline bilirubin as independent predictors for chemotherapy-induced neutropenia [25]. Using prediction models of this type is considered to be capable of identifying patients who may need low relative dose intensities and may be at risk of FN.

Research studies have been published that investigate genetic risk factors for chemotherapy-induced NLEs in patients with breast cancer [26–29] and other types of tumor [30–32]. Most of the studies concerned have been retrospective candidate gene studies. The largest of them investigated FEC chemotherapy in approximately 1000 breast cancer patients and concluded that adding single nucleotide polymorphisms (SNPs) to clinical models for predicting FN might be able to improve the prediction of such events [28, 29]. One smaller study performed a genome-wide association study (GWAS) in 270 Asian patients with different types of solid tumor histology [32] and found that SNPs in *MCPH1* were predictive for chemotherapy-induced neutropenia or leukopenia.

The primary aim of the present study was to analyze the predictive value of genetic variants in genes associated with NLEs that were identified in a previous GWAS in lymphoblastoid cell lines [33] and additional candidate genes. Analyses were carried out separately for two sequential chemotherapies (three cycles of FEC followed by three cycles of docetaxel/gemcitabine; study aims 1a and 1b). As an exploratory aim, these SNPs were each additionally analyzed in relation to overall survival (study aim 2a) and disease-free survival (study aim 2b), both in the complete cohort and in subgroups based on intrinsic molecular subtypes.

## RESULTS

### Patient characteristics

A total of 1678 patients from the prospective phase III chemotherapy study SUCCESS were included in the analysis for predicting chemotherapy-induced grade 3 or grade 4 neutropenia on the basis of clinical variables and the 246 SNPs analyzed. The percentage of missing values for each predictive variable was < 1%, with the exception of human epithelial growth factor receptor 2 (HER2, 2%) and four SNPs with missing values between 1.4% and 2.7%. In all, 97.6% of all the SNPs had a call rate of more than 99%. The patient and tumor characteristics relative to adverse event status (study aim 1a) are shown in Table 1. The frequencies of grade 3 or 4 neutropenia or leukopenia were in the expected ranges, at around 50% (Supplementary Tables 1 and 2). The corresponding rate of G-CSF administration is shown in Supplementary Table 3 and was low, at about 7% of patients, before the occurrence of neutropenic or leukopenic events. In relation to survival, 186 cases of progression and 102 deaths were observed. The median follow-up period was 4.9 years, both for overall survival and disease-free survival.

**Table 1:** Patient and tumor characteristics relative to adverse event status (neutropenia or leukopenia within the first three cycles, study aim 1a), showing mean and standard deviation (SD) for age, body mass index (BMI), and body surface area (BSA), and frequencies and percentages for all other characteristics

Characteristic	Adverse event = yes		Adverse event = no	
	Mean or n	SD or %	Mean or n	SD or %
Age	54.2	10.5	52.6	10.5
BMI	25.9	4.8	26.5	5.3
BSA	1.8	0.2	1.8	0.2
Tumor stage				
pT0	1	0.1	0	0.0
pT1	354	41.7	362	43.6
pT2	438	51.7	407	49.0
pT3	45	5.3	49	5.9
pT4	10	1.2	12	1.4
Nodal status				
pN0	280	33	277	33.4
pN+	568	67	553	66.6
Tumor type				
Ductal	692	81.6	683	82.3
Lobular	97	11.4	96	11.6
Other	59	7.0	51	6.1
Grade				
G1	51	6.0	38	4.6
G2	406	47.9	419	50.5
G3	391	46.1	373	44.9
Estrogen receptor status*				
Negative	270	31.8	240	28.9
Positive	578	68.2	590	71.1
Progesterone receptor status^†^				
Negative	318	37.5	295	35.5
Positive	530	62.5	535	64.5
HER2 status^‡^				
Negative	654	77.1	625	75.3
Positive	194	22.9	205	24.7

*Estrogen receptor (ER) positivity was defined by the study site. At the time of data collection, a cut-off point of 10% immunohistochemically positively stained cells was usually considered to be positive.

† Progesterone receptor (PR) positivity was defined by the study site. At the time of data collection, a cut-off point of 10% immunohistochemically positively stained cells was usually considered to be positive.

‡ HER2 status was considered to be positive if a staining of 3+ was achieved on immunohistochemical staining or there was a positive fluorescent *in situ* hybridization (FISH) test with a staining of 2+.

**Table 2:** SNPs with the lowest *P* values associated with adverse events (AEs) in chemotherapy cycles 1, 2, and 3

SNP	Chr	Position	Closest gene(s)	MAFAE yes	MAFAE no	Raw *P* value	Corrected *P* value
rs12050587	15	55335330	*PIGB*	26.8	21.3	1.0 × 10^–4^	0.03
rs11636687	15	55312954	*RAB27A | PIGB*	20.5	15.6	1.2 × 10^–4^	0.03
rs9514827	13	108267055	*ABHD13 | TNFSF13B*	28.0	32.6	2.9 × 10^–3^	0.70
rs4261468	15	55263404	*RAB27A*	24.1	20.2	4.6 × 10^–3^	1.00
rs2290344	15	55327598	*PIGB*	13.1	10.2	6.4 × 10^–3^	1.00
rs12050885	15	55266426	*RAB27A*	15.2	12.2	8.2 × 10^–3^	1.00
rs8024695	15	55347107	*PIGB*	13.3	10.7	1.9 × 10^–2^	1.00
rs4896870	6	146506145	*GRM1 | RAB32*	11.7	9.3	2.6 × 10^–2^	1.00
rs2595500	11	6941934	*ZNF215*	19.1	22.1	3.6 × 10^–2^	1.00
rs2607659	8	102227775	*RRM2B*	46.8	50.1	4.7 × 10^–2^	1.00

AE, adverse event; Chr, chromosome; MAF, minor allele frequency; SNP, single nucleotide polymorphism.

The table lists minor allele frequencies (MAFs) as well as raw and corrected *P* values resulting from comparison between the genetic and clinical logistic regression models (“Gene1 | Gene2” indicates the two genes between which the SNP is located).

### Prediction of neutropenia or leukopenia in the first three cycles (study aim 1a)

The preliminary logistic regression analyses for predicting NLEs with clinical parameters showed that the continuous predictors age and body surface area (BSA) both fitted best as linear predictors. The clinical regression model indicated that the risk of having an adverse event increases with increasing age (OR per year of increase and fixed BSA, 1.02; 95% CI, 1.01 to 1.03) and decreasing BSA (OR per unit increase and fixed age, 0.49; 95% CI, 0.27 to 0.89).

In addition to these clinical parameters, logistic regression models were constructed that included the SNPs. The ten SNPs with the lowest *P* values are listed in Table 2, along with the minor allele frequencies. Two SNPs maintained statistical significance after correction of *P* values: rs12050587 (corrected *P* = 0.03, likelihood ratio test) and rs11636687 (corrected *P* = 0.03, likelihood ratio test). These SNPs were significant predictors in addition to age and BSA. The OR for rs12050587 was 1.38 per minor allele (95% CI, 1.17 to 1.62), and it was 1.43 (95% CI, 1.19 to 1.71) per minor allele for rs11636687. Sensitivity analyses with G-CSF administration as a predictor yielded similar results for cycle 1. The corrected *P* values for rs12050587 and rs11636687 were 0.056 and 0.03, respectively. No significant *P* values were seen for any SNPs in cycles 2 and 3.

The area under the receiver operating curve (AUC) in the clinical regression model was 0.554, whereas the AUCs in the models that included rs12050587 and rs11636687 were 0.575 and 0.572, respectively. The net reclassification improvement (NRI) for the rs12050587 model was 0.15, showing a moderate increase in performance with addition of the SNP information. Prediction improved for 22% of controls, but was reduced for 8% of cases. The NRI for the rs11636687 model was 0.14. The genotypes of both SNPs were in linkage, with an *r*^2^ of 0.67.

Internal validation showed that the clinical model is slightly overfitted, with a validated AUC of 0.550. The validated AUC of 0.556 for the genetic model with the smallest *P* value indicated some overfitting of the rs12050587 model. The validated NRI was 0.08.

### Prediction of neutropenia or leukopenia in the last three cycles (aim 1b)

As described above for the first three cycles, the continuous predictors age and BSA fitted best, both as linear predictors. The clinical prediction model (AUC = 0.51) is no more useful than the null model without any predictors. Age and BSA thus did not predict the adverse events noted in the last three cycles.

After correction for multiple testing, none of the SNPs added any value for predicting white blood cell toxicity in the last three cycles (lowest *P* value 0.26 after correction for multiple testing, likelihood ratio test). Sensitivity analyses with G-CSF administration as a predictor also showed no statistical significance for any SNP in any cycle.

### Prediction of prognosis (aims 2a and 2b)

The analyses of overall survival and disease-free survival did not identify any significant prognostic effects for any of the SNPs examined, after correction for multiple testing in either the molecular subtypes or across all patients. The minor allele frequencies and raw and corrected *P* values for both analyses are shown in Supplementary Tables 4 and 5. All of the corrected *P* values for disease-free survival equaled 1, except for rs12640749 (corrected *P* = 0.35). In the analysis of overall survival, the SNPs rs6946062 (corrected *P* = 0.48) and rs10820726 (corrected *P* = 0.71) had the lowest *P* values.

## DISCUSSION

This study found suggestive evidence that the SNPs rs12050587 and rs11636687, which are in close linkage to each other, are associated with a risk for developing a neutropenic or leukopenic event (NLE) after FEC chemotherapy. The two SNPs are located in the *PIGB* gene (phosphatidylinositol glycan anchor biosynthesis, class B). No other SNPs were found to be significant either in relation to predicting such adverse events or in relation to the prognosis, as an exploratory study aim.

There have been previous reports in studies with reasonable sample sizes that have investigated whether candidate gene SNPs have any influence on the occurrence of febrile neutropenia (FN) or NLE. A study including just over 1000 breast cancer patients who received FEC chemotherapy examined 59 SNPs in 28 genes that had previously been reported to be associated with neutropenic events or had been described as playing a key role in metabolizing these three chemotherapeutic agents [28]. One SNP (rs4148350) in the *ABCC1* gene, also known as *MRP1* (multidrug resistance–associated protein 1), was associated with febrile neutropenia (the primary study aim), with an OR of 1.80 (95% CI, 1.11 to 2.86) for a heterozygous genotype. The homozygous genotype was quite rare (0.06%) and yielded an OR of 21.14 (95% CI, 3.07 to 416.57) [28]. With regard to NLE (the secondary study aim), the study reported that rs4148350 and rs246221 in *ABCC1* and rs76688282 in *UGT2B7* were significantly associated with prolonged grade 3–4 or deep neutropenia [28].

No previous studies have reported on the SNPs analyzed in the present study, in which *PIGB* was found to be associated with neutropenic or leukopenic events after chemotherapy with FEC. There are several possible explanations for why no genotype effects on gemcitabine toxicity were found. All of the patients were scheduled for a total of six chemotherapy cycles, of which the first three were the same in all patients and consisted of FEC, while the last three cycles consisted of either docetaxel or docetaxel and gemcitabine. The effects of a lower bone marrow reserve after the first three cycles might thus have had an influence on the patients’ response to the fourth cycle, which would then differ from the response in the absence of any prior chemotherapy. In addition, in accordance with the study protocol, G-CSF administration was not indicated for primary prophylaxis, although patients who had had NLEs in previous cycles were required to be treated with G-CSF. The proportion of patients who were treated with G-CSF might therefore have been quite different in the last three cycles than in the first three. Performing sensitivity analyses for each single cycle and including G-CSF use as a predictor showed that the predictive effect of the SNPs was largest only in the first cycle and that it was not present in a group of patients who had previously undergone chemotherapy.

No statistically significant associations were identified with regard to the effect of the genotypes on NLEs in the last three cycles, including the randomization arm. This need not necessarily imply that there is no effect, but the effect might be influenced by the factors mentioned above or might not be evident due to a lack of statistical power.

Although there have been a few reports describing better survival in patients who experienced a neutropenic event after chemotherapy [34, 35], no such association was found in the present study in relation to the exploratory study aims of overall survival and disease-free survival. This might not be unusual, as there have also been other studies reporting poorer survival in patients who did not receive the full dosage of the planned chemotherapy, due to neutropenia.

*PIGB* is of special interest in view of the findings of a previous study by our group of gemcitabine toxicity in lymphoblastoid cell lines [36]. The study found that not only *PIGB* genotypes (seven SNPs) but also *PIGB* gene expression were associated with the response to gemcitabine treatment. In addition, genotypes were associated with gene expression [36] with a clear *cis* expression quantitative trait locus (eQTL) association in lymphoblastoid cell lines. rs12050687 showed lower gene expression for the rare genotype. Both the rare genotype and lower expression resulted in a higher inhibitory concentration of 50% (IC_50_) [36]. In the present study, none of the SNPS in the *PIGB* region correlated with hematotoxicity during the last three chemotherapy cycles. This might be due to the reasons mentioned above — previous chemotherapy and administration G-CSF. However, the rare alleles of the SNPs in *PIGB* were shown to be indicative of more frequent neutropenic and leukopenic events during treatment with FEC.

The effects of the genotype showed a different trend with FEC chemotherapy than in previous *in vivo* results with regard to gemcitabine [36]. This may suggest that the different chemotherapies are associated with different interactions with white blood cells. The fact that these were not seen in the present study might be a consequence of low power, or might be due to the combination therapy with docetaxel. Although this is the largest study yet conducted in this connection, the differential effects of genotypes on the toxicity of different chemotherapies will have to be explored in further studies.

The validated AUC for the SNP model was slightly better than the validated AUC for the clinical model. To assess this effect, one should be aware that increases in AUC are often very small, even for markers that are strongly associated with the outcome [37–39]. Because of this, reclassification measures such as the NRI have been developed to allow closer analysis of groups of patients who might be able to benefit from advanced prediction models. The Supplementary Materials provided here may be consulted for further discussion of model performance.

The present study has some limitations. Most of the SNPs were selected in relation to their ability to predict gemcitabine toxicity in cell culture models with lymphoblastoid cell lines. The analysis in relation to this study aim did not confirm this observation, either for the overall group of patients or for patients in a specific randomization arm. The result was therefore unexpected and needs to be interpreted with care. However, the *P* value was low enough for significance to be maintained after correction for multiple testing. The study aim was prespecified in the original subprotocol, but the analyses were carried out on a subset of the main study. There were no differences between this subset and the main study in relation to the patient characteristics. With regard to the assessment of NLEs, it needs to be borne in mind that blood monitoring was carried out in accordance with common clinical practice. However, the documentation was prospective and the highest toxicity grade had to be documented at the time point of the next chemotherapy cycle or at the final assessment of chemotherapy.

## MATERIALS AND METHODS

### Patients and treatment

The patients included in this analysis were selected from the multicenter SUCCESS-A study [40–42], for which patients were eligible if they had invasive breast cancer (pT1–3) with a high risk of recurrence — defined as tumors that were either node-positive, large (≥ pT2 and grade 3), or with negative hormone receptor status. The SUCCESS-A study was conducted in 251 study centers in all regions of Germany. All of the study centers participated in the prospectively designed translational research subprotocols. The main study and all of the prespecified translational research projects, including the one reported here, were approved by all of the ethics committees responsible and were conducted in accordance with the Declaration of Helsinki. All of the patients provided written informed consent.

Patients in the SUCCESS-A study were treated with three cycles of fluorouracil, epirubicin, and cyclophosphamide (500/100/500 mg/m^2^; FEC) followed by three cycles of docetaxel (100 mg/m^2^) every three weeks (q3w), versus three cycles of FEC followed by three cycles of gemcitabine (1000 mg/m^2^ d1,8) and docetaxel (75 mg/m^2^) q3w. The main results of the study have been reported elsewhere [43]. Following the completion of chemotherapy, the patients underwent random assignment once again to receive either 2 or 5 years of zoledronic acid. Premenopausal hormone receptor–positive women received tamoxifen alone or in combination with goserelin for 2 years if they were younger than 40 years of age. Postmenopausal patients were treated with tamoxifen for 2 years, followed by anastrozole for 3 years.

The primary surgery consisted of either breast conservation or mastectomy, leading to R0 resection in all cases. Sentinel-node dissection (SND) was performed in all cN0 patients (with SND as the only axillary intervention), followed by complete axillary node dissection in patients with positive sentinel nodes. The cN1 patients primarily received axillary node dissection. Radiotherapy was performed in accordance with national guidelines [44, 45] and was used in all patients who received breast-conserving treatment.

### Clinicopathologic information and follow-up

For assessment of leukopenia or neutropenia, the patients were monitored in accordance with common clinical practice and were documented in accordance with the National Cancer Institute Common Terminology Criteria for Adverse Events (NCI-CTCAE), version 2. The mean periods from the chemotherapy cycle to the occurrence of documented neutropenia or leukopenia were 11 days during the first three cycles and 8 days during the last three cycles. Documentation of G-CSF administration was documented for each cycle of chemotherapy given. Data for G-CSF administration are presented in Supplementary Table 1.

For survival data, the patients were followed up at the study sites at 3-month intervals for the first 3 years and every 6 months thereafter. The follow-up included a clinical examination (at each visit), mammography (every 6 months) and symptom-driven examinations if necessary. The quality of the data was ensured by electronic data management, including automated plausibility checks and regular monitoring visits to the study site by an independent clinical research organization (Alcedis GmbH, Giessen, Germany) and a data monitoring committee (DMC).

### Biomaterial sampling and patient selection

**Figure 1:**
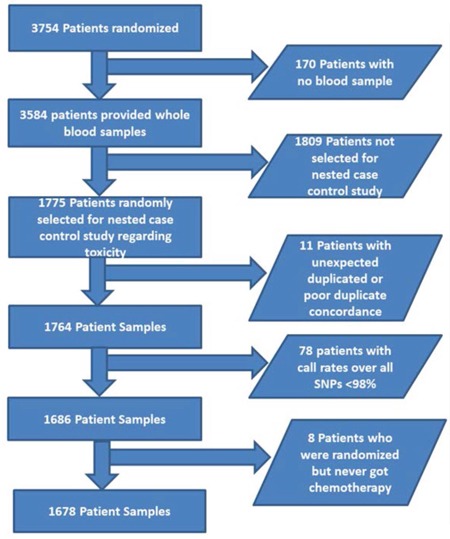
Patient selection chart

A total of 3754 patients underwent random assignment between September 2005 and March 2007. Whole-blood samples were retrieved from 3584 patients at the time point of randomization. To build a nested case–control study, 887 patients were randomly selected from the group of patients with grade 3 or 4 NLE (cases) and 888 patients from the group of patients without grade 3 or 4 NLE (controls) in the first three cycles of the chemotherapy, resulting in a sample size of 1775 patients for genotyping. Eleven patients were excluded because of duplicate issues, and 78 patients were excluded because less than 98% of all genotyped SNPs could be called. Finally, eight additional patients who had undergone randomization but never started chemotherapy treatment were excluded. The final sample size for this study was therefore 1678. The flow chart of the patient selection process is shown in Figure 1.

### SNP selection and lymphoblastoid GWAS

Our group has previously published a GWAS of gemcitabine pharmacogenomics using 180 human lymphoblastoid cell lines (LCLs) [33]. In the current study, the top 200 SNPs associated with gemcitabine IC_50_ values in LCLs were selected, plus 153 tag SNPs for genes in the gemcitabine metabolism and activation pathway [33]. In addition, 31 candidate SNPs associated with breast cancer prognosis on the basis of literature reports were also selected, but these were not included in this analysis and are not reported on here.

### Genotyping

Genotyping of these 384 SNPs was performed as part of an Illumina GoldenGate custom panel, using a standard operating procedure based on the manufacturer's protocol [46] (Illumina Inc., San Diego, California, USA). The BeadArray microarrays were scanned and the fluorescent signals were analyzed using the GenomeStudio software program (Illumina, Inc.) for automated genotype clustering and calling.

Genotyping was considered to be successful for 364 SNPs after a manual review of the clustering. SNPs were excluded from analysis in accordance with the following hierarchical criteria: candidate SNPs selected for prognostic analyses (excluding 31 SNPs); any SNP for which the overall call rate was < 95% (excluding three SNPs); any SNP for which the *P* value for departures from Hardy–Weinberg proportions for controls was < 0.005 (excluding one SNP); and SNPs with a minor allele frequency (MAF) < 0.1, excluded for power reasons (excluding 83 SNPs). The total number of SNPs included in the analysis was 246.

### Statistical methods

The four study aims were analyzed separately — two concerned with prediction of NLEs (aims 1a and 1b) and two concerned the with overall survival and disease-free survival (aims 2a and 2b). The Supplementary Methods section may be consulted for a precise description.

Logistic regression analyses were carried out to investigate the predictive value of each SNP relative to the occurrence of at least one grade 3 or 4 NLE within the first three chemotherapy cycles (adverse event status = “yes”) versus the nonoccurrence of these events (adverse event status = “no”), in addition to clinical parameters (aim 1a). Logistic regression analyses were also performed for the outcome of an NLE during the last three cycles, in order to study overall and treatment-specific associations (docetaxel vs. docetaxel and gemcitabine in the last three cycles) between SNP and outcome (aim 1b). Cox regression analyses were carried out to explore the overall and molecular subtype–specific prognostic effect of each SNP with regard to overall survival (aim 2a) and disease-free survival (aim 2b), in addition to clinical parameters.

Patients with any outcome variables lacking were excluded. Missing clinical predictor values were imputed using single “best guesses.” Continuous predictors were used as natural cubic spline functions to describe nonlinear effects. In each analysis, a (logistic or Cox) regression model with clinical predictors but no SNP information was set up as a reference model. For each SNP, a regression model was fitted with the SNP (ordinal; 0, 1, or 2 minor alleles), the predictors of the clinical model and, if necessary, interaction terms for subgroup-specific results. The genetic regression models were compared with the clinical regression model using the likelihood ratio test. A significant test result means that the SNP has predictive value independently of the clinical characteristics. The *P* values for these likelihood ratio tests (one test for each SNP) were corrected using the Bonferroni–Holm method, to address the problem of multiple testing. If a corrected *P* value was significant, then the genetic regression model was applied to calculate the overall effect and, if specified, subgroup-specific effects in terms of odds ratio (OR) or hazard ratio (HR) per minor allele of the SNP were adjusted for the clinical parameters.

The predictive performance of the logistic regression models in terms of discrimination of cases and controls was assessed using the AUC and the NRI. A bootstrap-based internal validation procedure was performed to obtain nearly unbiased estimates of model performance [47].

Sensitivity analyses were conducted to take account of the influence of G-CSF on the outcome NLE. For each cycle (from 1 to 6), logistic regression analyses were repeated with G-CSF administration (yes/no) as an additional predictor. G-CSF administration was not taken into account in the main analyses, since three cycles were summarized there and G-CSF administration can only be assigned to a single cycle.

All of the tests were two-sided, with the significance level set at 0.05. *P* values were corrected as described above only within the four analyses (1a, 1b, 2a, 2b), but not across the analyses. Calculations were carried out using the R system for statistical computing, version 3.0.1.

## CONCLUSIONS

This study has shown that genetic variation in the *PIGB* gene is associated with neutropenic or leukopenic events in patients who are treated with FEC chemotherapy. It remains to be determined whether and in what ways this finding could potentially be incorporated into predictive models for clinical use. Further validation in other study cohorts is warranted, as this is the first report that has described these variants in a clinical study.

## SUPPLEMENTARY MATERIALS TABLES



## Abbreviations

*ABCC1*: ATP binding cassette subfamily C member 1 (gene)
AUC: area under the curve
BSA: body surface area
CI: confidence interval(s)
DMC: data monitoring committee
eQTL: expression quantitative trait locus
FEC: 5-fluorouracil, epirubicin, and cyclophosphamide (chemotherapy)
FN: febrile neutropenia
G-CSF: granulocyte colony-stimulating factor
GWAS: genome-wide association study
HER2: human epithelial growth factor receptor 2
HR: hazard ratio
IC_50_: inhibitory concentration of 50%
LCL: lymphoblastoid cell line
MAF: minor allele frequency
*MCPH1*: microcephalin 1 (gene)
*MRP1*: multidrug resistance–associated protein 1 (gene)
NCI-CTCAE: National Cancer Institute Common Toxicity Criteria for Adverse Events
NLE: neutropenic or leukopenic event
NRI: net reclassification improvement
OR: odds ratio
*PIGB*: phosphatidylinositol glycan anchor biosynthesis class B (gene)
SND: sentinel-node dissection
SNP: single nucleotide polymorphism
*UGT2B7*: UDP glucuronosyltransferase family 2 member B7 (gene).
