# Supplementary material for: Clinical validation of genetic variants associated with in vitro chemotherapy-related lymphoblastoid cell toxicity
Source: Oncotarget. 2017 May 9;8(44):78133–43. doi: 10.18632/oncotarget.17726 (PMC5652844; doi:10.18632/oncotarget.17726)
Supplement: Supplementary file 1 [file oncotarget-08-78133-s001.pdf]

Clinical validation of genetic variants associated with *in vitro* chemotherapy-related lymphoblastoid cell toxicity

SUPPLEMENTARY METHODS

Online statistical considerations

The four study aims were analyzed separately, concerned with prediction of neutropenic or leukopenic events (aims 1a and 1b) and two concerned with prognosis of overall and disease-free survival (aims 2a and 2b). For these analyses, patients with missing outcome variable were excluded. Then 96.9% of all patients had complete patient and tumor characteristics, Missing clinical predictor values were imputed using single "best guesses" (median value of continuous predictors, the most common value of categorical or ordinal categorical predictors) based on non-missing data across all subjects. Continuous predictors were used as natural cubic spline functions to describe non-linear effects. The degrees of freedom (d.f.) of each predictor was determined by first fitting several simple cubic spline regression models (logistic or Cox regression, depending on the study aim) which differ from each other by the d.f. (from 1 to 3) and then choosing the d.f. which optimizes the Akaike information criterion.

Logistic regression analyses were carried out to investigate the predictive value of each SNP relative to the occurrence of at least one grade 3 or 4 NLE within the first three chemotherapy cycles (adverse event status = "yes") versus the nonoccurance of these adverse events within the first three chemotherapy cycles (adverse event status = "no"), in addition to clinical parameters (study aim 1a). First, a clinical model with age at diagnosis (continuous) and body surface (BSA; continuous) as predictors was set up as reference model. For each SNP, a logistic regression model with the SNP (ordinal; 0, 1, or 2 minor alleles) and the predictors of the clinical model was fitted. The genetic regression models were compared with the clinical regression model using the likelihood-ratio test. A significant test result means that the SNP has predictive value independently of the clinical characteristics. The *P* values for these likelihood ratio tests (one test for each SNP) were corrected using the Bonferroni-Holm method, to address the problem of multiple testing. The Bonferroni-Holm method is a less conservative refinement of the classical Bonferroni correction and similar to that, it controls the family-wise error rate [1, 2]. That is, the probability of at least one false-positive SNP among all SNPs whose corrected *P* values were significant is less or equal to the significance level. If a corrected *P* value was significant, then the odds ratio (OR) per minor allele of the SNP adjusted for the clinical parameters was calculated by applying the genetic regression model.

The predictive performance of the regression models in terms of discrimination of cases and controls was assessed using the area under the receiver operating curve (AUC). The AUC is easy to understand but it might be quite insensitive at the comparison of a genetic model with the clinical model if the SNP is effective only for a small part of the population [3]. To overcome this disadvantage a net reclassification improvement (NRI) was proposed, originally only category-based and later also defined as a continuous measure directly applicable for case-control studies [4, 5]. Roughly speaking, the continuous NRI is the proportion of cases or controls which are correctly given a higher or lower predicted probability of being a case by the genetic model than by the clinic model corrected by wrongly given lower or higher probabilities.

The AUC and NRI values may be too optimistic as model fitting and performance measuring were carried out on the same data set. A bootstrap-based internal validation procedure was performed to obtain nearly unbiased estimates of model performance [6]. Briefly, 1,000 bootstrap samples were taken with replacement from the original data set. On each bootstrap sample, the whole model building process (determining d.f. for continuous predictors, fitting genetic model, likelihood ratio test, correction of *P* values) was done, and the SNP with the smallest *P* value was selected to work on with. In case of significance, AUCs for the clinical and the genetic model and NRI were calculated on the bootstrap sample (denoted AUCboot and NRIboot), and the bootstrap model was applied to the original to get AUCs and NRI there (denoted AUCorig and NRIorig). The difference between the AUCboot and AUCorig is the "optimism" of the AUC. The optimism of the NRI is defined in the same way. Averaging the optimisms over all bootstrap sample yields the general optimism for AUC and NRI, respectively. The differences AUC minus its averaged optimism for the clinical as well as the genetic model and the difference NRI minus its averaged optimism are nearly unbiased ("honest") estimates of the expected value of the AUC and NRI for future cases and controls which do not belong to this study.

A similar regression analysis was performed for the outcome adverse events leukopenia or neutropenia within the last three chemotherapy cycles (yes vs. no) to study the overall and treatment-specific (docetaxel in the last three cycles vs. docetaxel and gemcitabine in the

last three cycles) association between SNP and outcome (study aim 1b). A clinical logistic regression model with the predictors age, BSA and treatment arm was compared with an extended regression model with the SNP, the clinical predictors and the interaction between SNP and randomization arm using a likelihood ratio test. The *P* values were corrected as described above. If a corrected *P* value was significant, then further analyses were planned. The genetic model was compared with a reduced genetic model without interaction term using a likelihood ratio test to discover whether the association between SNP and outcome was treatment-specific.

Sensitivity analyses were conducted to consider the influence of G-CSF on the outcome NLE. For each cycle (from 1 to 6), logistic regression analyses were repeated with G-CSF intake (yes/no) as additional predictor. G-CSF intake was not regarded in the main analyses, because three cycles were summarized there and G-CSF intake can only be assigned to a single cycle.

Overall survival (OS) was defined as the time interval from the date of diagnosis to the date of death or the date of censoring. Patients who were lost to follow-up were censored at the last date they were known to be alive. Progression-free survival (PFS) was defined as the time interval from the date of diagnosis to the date of first progression (distant metastasis, local recurrence, or death) or the date of censoring. Patients who were lost to follow-up were censored at the last date they were known to be distant metastasis-free and local recurrence-free.

An analysis strategy similar to that of the adverse events was applied both to OS (study aim 2a) and to PFS (study aim 2b). For each aim, a clinical Cox proportional hazards regression model with the established predictors age at diagnosis (continuous), BMI (continuous), pT (ordinal), ER (positive vs. negative), PR (positive vs. negative), HER2 (positive vs. negative), grading (ordinal; G1, G2, G3), nodal status (positive vs. negative), and histology (ductal, lobular, other) as main effects but without any SNP variables was fitted as reference model. For each SNP a Cox model was fitted with the SNP and the established predictors as main effects, and the interactions between SNP and ER, PR, HER2, and grading, respectively, as additional effects. These interaction terms were included to get specific results for intrinsic molecular subtypes. Comparisons of clinic and interaction models and subsequent *P* value corrections were performed. In case of significance, further analyses were planned to show whether the association between SNP and outcome varied between subgroups. The proportional hazards assumptions in the SNP models were checked using the Grambsch and Therneau method [7].

All of the tests were two-sided with significance level 0.05. *P* values were corrected as described above only within the four analyses (1a, 1b, 2a, 2b) but not across the analyses. Calculations were carried out using the R system for statistical computing (version 3.0.1; R Development Core Team, Vienna, Austria)

Online statistical discussion

The predictive performance of the regression models was internally validated using a bootstrap-based method. The clinical model was not overfitted, but the genetic models were overfitted to a certain amount. The reasons for these results may be the following: The clinical model was quite simple having only two, predefined predictors. The SNP predictor of the final genetic model, however, was selected out of several hundred SNPs, some of those true effects were overestimated by the regression model, others were underestimated. It is the “winner’s curse” that there are above-average many overestimated SNPs among the top-ranked SNPs. The validation process corrected a possible over-estimation. The validated AUC of the SNP model was slightly better than the validated AUC of the clinical model. To assess this effect, one should know that the increase in AUC is often very small even for markers which are strongly associated with the outcome [8–10]. Because of this, reclassification measures such as the NRI were developed to have closer look on patient groups which could benefit from advanced prediction models.

Strictly speaking, we did not validate the final model but the model building procedure which leads to a final model. The difference is important: The whole data set was used to select the significant SNP rs12050587. If the whole data set was divided into training and validation data and the genetic model with that SNP was fitted on the training data set and applied on the validation data to measure the performance (e.g. AUC), then the performance measure would be biased because all of the patients in the validation data were previously used to select the SNP. Unfortunately, this mistake is committed many times in published papers, and the reader should be cautious when he or she compares our results with others’. Instead, all model building processes has to be done only in the training data to get a model which can then be applied on unused data. Repetitions of validation steps may lead to various final SNPs. Of course, the best internal validation cannot replace an external validation of our findings in future studies.

REFERENCES

1. Holm S. A simple sequentially rejective multiple test procedure. Scand J Stat. 1979; 6:65-70.
2. Shaffer JP. Multiple hypothesis-testing. Annu Rev Psychol 1995; 46:561-84. doi: 10.1146/annurev. psych.46.1.561.
3. Gerds TA, Cai T, Schumacher M. The performance of risk prediction models. Biom J. 2008; 50:457-79. doi: 10.1002/bimj.200810443.

4. Pencina MJ, D'Agostino RB Sr, D'Agostino RB Jr, Vasan RS. Evaluating the added predictive ability of a new marker: from area under the ROC curve to reclassification and beyond. Stat Med. 2008; 27:157-72; discussion 207-12. doi: 10.1002/sim.2929.
5. Pencina MJ, D'Agostino RB Sr, Steyerberg EW. Extensions of net reclassification improvement calculations to measure usefulness of new biomarkers. Stat Med. 2011; 30:11-21. doi: 10.1002/sim.4085.
6. Harrell FE Jr, Lee KL, Mark DB. Multivariable prognostic models: issues in developing models, evaluating assumptions and adequacy, and measuring and reducing errors. Stat Med. 1996; 15:361-87. doi: 10.1002/(SICI)1097-0258(19960229)15:4<361::AID-SIM168>3.0.CO;2-4.
7. Grambsch PM, Therneau TM. Proportional hazards tests and diagnostics based on weighted residuals. Biometrika. 1994; 81:515-26.
8. Pepe MS, Janes H, Longton G, Leisenring W, Newcomb P. Limitations of the odds ratio in gauging the performance of a diagnostic, prognostic, or screening marker. Am J Epidemiol. 2004; 159:882-90.
9. Ware JH. The limitations of risk factors as prognostic tools. N Engl J Med. 2006; 355:2615-7. doi: 10.1056/NEJMp068249.
10. Wang TJ, Gona P, Larson MG, Tofler GH, Levy D, Newton-Cheh C, Jacques PF, Rifai N, Selhub J, Robins SJ, Benjamin EJ, D'Agostino RB, Vasan RS. Multiple biomarkers for the prediction of first major cardiovascular events and death. N Engl J Med. 2006; 355:2631-9. doi: 10.1056/NEJMoa055373.

Supplementary Table 1: Leukopenic or neutropenic events within first three cycles of chemotherapy

**Adverse event**		**All grades** *N*	**All grades** %	**Grade 3 and 4** *N*	**Grade 3 or 4** %
Neutropenia without febrile neutropenia	No	1057	63.0	1190	70.9
	Yes	621	37.0	488	29.1
Leucopenia	No	501	29.9	988	58.9
	Yes	1177	70.1	690	41.1
Febrile neutropenia	No	1587	94.6	1644	98.0
	Yes	91	5.4	34	2.0
Neutropenia or leucopenia	No	424	25.3	830	49.5
	Yes	1254	74.7	848	50.5

Supplementary Table 2: Neutropenic or leukopenic events within last three cycles of chemotherapy according to treatment arm

**Adverse event**		**Doc/Gem all grades *N***	**Doc/Gem all grades %**	**Doc all grades *N***	**Doc all grades %**	**Doc/Gem grade 3 and 4 *N***	**Doc/Gem grade 3 and 4 %**	**Doc grade 3 and 4 *N***	**Doc grade 3 and 4 %**
Neutropenia without febrile neutropenia	No	524	64.6	542	66.5	594	73.2	582	71.4
	Yes	287	35.4	273	33.5	217	26.8	233	28.6
Leucopenia	No	198	24.4	278	34.1	361	44.5	403	49.4
	Yes	613	75.6	537	65.9	450	55.5	412	50.6
Febrile neutropenia	No	749	92.4	746	91.5	779	96.1	772	94.7
	Yes	62	7.6	69	8.5	32	3.9	43	5.3
Neutropenia or leucopenia or febrile neutropenia	No	158	19.5	243	29.8	357	44.0	361	44.3
	Yes	653	80.5	572	70.2	454	56.0	454	55.7

Supplementary Table 3: Use of G-CSf over all cycles and neutropenic or leukopenic events (NLE) grade 3 or 4

Cycle	G-CSF use	All patients *N* (%)	Patients wihout NLE *N* (%)	Patients with NLE *N* (%)
Cycle 1	No	1477 (88.2)	937 (93.3)	540 (80.6)
	Yes	197 (11.8)	67 (6.7)	130 (19.4)
Cycle 2	No	1390 (83.0)	1020 (86.7)	370 (76.8)
	Yes	268 (16.0)	156 (13.3)	112 (23.2)
Cycle 3	No	1336 (79.8)	1025 (84.6)	311 (72.0)
	Yes	308 (18.4)	187 (15.4)	121 (28.0)
Cycle 4	No	1088 (65)	727 (74.6)	361 (55.9)
	Yes	533 (31.9)	248 (25.4)	285 (44.1)
Cycle 5	No	957 (57.1)	645 (63.7)	312 (54.1)
	Yes	633 (37.8)	368 (36.3)	265 (45.9)
Cycle 6	No	961 (57.4)	693 (62.7)	268 (58.9)
	Yes	599 (35.8)	412 (37.3)	187 (41.1)

Supplementary Table 4: SNPs with smallest *P* values for the association between genotype and progression-free survival

SNP	Chromosome	Position	MAF	Raw *P* value	Corrected *P* value
rs12640749	4	173346075	43.7	1.4×10^{-3}	0.35
rs575156	10	29209280	36.6	8.9×10^{-3}	1.00
rs7950019	11	18257365	33.2	9.0×10^{-3}	1.00
rs8140044	22	39710977	14.0	1.1×10^{-2}	1.00
rs815437	3	55705332	38.8	1.9×10^{-2}	1.00
rs10820726	9	104770036	19.8	2.3×10^{-2}	1.00
rs17019442	1	212600998	10.1	2.5×10^{-2}	1.00
rs2472476	9	104769675	38.4	2.5×10^{-2}	1.00
rs16941238	15	45269706	10.3	3.0×10^{-2}	1.00
rs2282791	5	136042041	44.4	3.2×10^{-2}	1.00

The minor allele frequencies (MAFs) as well as raw and corrected *P* values resulting from the comparison between the genetic and the clinical Cox regression model are shown.

Supplementary Table 5: SNPs with smallest *P* values for the association between genotype and overall survival

SNP	Chromosome	Position	MAF	Raw *P* value	Corrected *P* value
rs6946062	7	33043495	42.6	2.0×10^{-3}	0.48
rs10820726	9	104770036	19.8	2.9×10^{-3}	0.71
rs2472476	9	104769675	38.4	4.4×10^{-3}	1.00
rs9458486	6	162206258	22.1	8.4×10^{-3}	1.00
rs10761082	9	104753888	38.7	8.5×10^{-3}	1.00
rs9867082	3	86377074	10.9	2.8×10^{-2}	1.00
rs4789636	17	74202700	44.5	3.2×10^{-2}	1.00
rs2172820	8	15468159	27.0	3.6×10^{-2}	1.00
rs10993751	9	90900359	44.4	4.5×10^{-2}	1.00
rs12148896	15	84891963	49.4	5.2×10^{-2}	1.00

The minor allele frequencies (MAFs) as well as raw and corrected *P* values resulting from the comparison between the genetic and the clinical Cox regression model are shown
